# Amplicon-Based NGS Panels for Actionable Cancer Target Identification in Follicular Cell-Derived Thyroid Neoplasia

**DOI:** 10.3389/fendo.2020.00146

**Published:** 2020-03-24

**Authors:** Majbritt Busk Madsen, Katalin Kiss, Finn Cilius Nielsen, Finn Noe Bennedbæk, Maria Rossing

**Affiliations:** ^1^Center for Genomic Medicine, Rigshospitalet, Copenhagen University Hospital, Copenhagen, Denmark; ^2^Department of Pathology, Rigshospitalet, Copenhagen University Hospital, Copenhagen, Denmark; ^3^Department of Endocrinology, Herlev University Hospital, Herlev, Denmark

**Keywords:** follicular cell-derived thyroid neoplasia, FFPE-preserved DNA, next-generation sequencing, somatic mutation profile, somatic variant classification

## Abstract

Follicular cell-derived thyroid cancers are heterogenous and morphological classification is a complex and highly specialized task. Hence, identification of somatic alterations could provide insights to tumor biology and serve as an add-on diagnostic tool. Furthermore, results from these add-on tools could point in the direction of a more personalized treatment strategy. In the present study we set out to identify and validate the somatic mutation profile in a sample-set of follicular cell-derived thyroid neoplasia. One-hundred-and-one archived formalin fixed paraffin embedded (FFPE) tissue samples from patients diagnosed with follicular cell-derived thyroid neoplasia were included, and upon DNA-extraction and qualitative measurements 99 samples were eligible for amplicon-based next-generation-sequencing. Libraries were generated using the TruSeq Amplicon Cancer Panel, followed by sequencing using a MiSeq. Upon data processing and variant filtering all variants were manually assessed to exclude false positive mutations in the final curated list. Moreover, hot-spot mutations were validated using an independent platform from Agilent. Each diagnostic group were correlated to mutation burden and individual mutations were classified according to recent guidelines for somatic mutation classification. Close to 100% of the archived FFPE samples were eligible for DNA-library preparation and amplicon sequencing based on DNA quality criterion. The distribution of mutations in the specific diagnostic groups resulted in a higher mutation frequency among the most dedifferentiated than in the groups with a more differentiated cell profile. Based on the distribution mutations across the samples and using hierarchical clustering, we generated four tentative mutational signatures; highly mutated tumors; tumors with mainly *NRAS* and *TP53* mutations; *BRAF* mutated tumors and tumors with none or single sporadic mutations. Future studies including more samples and follow-up data may amend these signatures, however our results imply that morphological classification of follicular cell derived thyroid neoplasia could be supplemented with a somatic mutational signature. Taken together, broad screening of the somatic alterations in FFPE tissue of thyroid neoplasia is comprehensible and essential for future identification of possible treatment targets and personalized medicine.

## Introduction

Thyroid cancer is the most frequent endocrine malignancy with an annual incidence of around 300.000 cases worldwide (1). Most of thyroid cancers are follicular cell-derived comprising about 95%, whereas the remaining 5% are the medullary thyroid carcinomas which originate from the parafollicular C-cells. The follicular cell-derived thyroid cancers can generally be classified into papillary thyroid cancer (PTC) 80–90%, follicular thyroid cancer (FTC) 10–15% (including minimally and widely invasive follicular thyroid cancer; miFTC and wiFTC, respectively), poorly differentiated thyroid cancer (PDC) and anaplastic thyroid cancer (ATC) ~3% (2, 3). The benign counterpart of FTCs is the follicular thyroid adenoma (FA) and the sole features separation FA from FTC are morphological: vascular and/or capsular invasion (4) making the preoperative differentiation and classification extremely challenging. Moreover, follicular cell-derived thyroid cancers are heterogenous which makes histopathological uniform classifications a highly specialized task (5). However, due to advances in molecular technologies, a deeper understanding of the pathophysiology, accompanied by genetic and epigenetic alterations of follicular cell-derived thyroid tumors has occurred (6–8). Whereas, the genomic landscape and oncogenic events in PTC have been well-portraited, the molecular spectrum of FTC is less well-known (9). Nevertheless, as oncogenic events determine the dedifferentiation of the follicular cell-derived cancers, a more extensive curation of somatic mutations within this heterogenous neoplasia, could be a valuable diagnostic tool and add to the existing morphological classification (3). Moreover, mutation profiling could help the preoperative diagnostic work-up and therapeutic decision making.

With the increasing number of biomarkers in cancer diagnostics, next-generation sequencing (NGS) is readily implemented in the diagnostic and clinical routine, playing an important role for personalized medicine. Several targeted gene-panels and sequencing platforms have been launched, but in broad they all offer multiple variant identification in a single assay from a small DNA-input, fast turn-around time and low-cost (10). However, there are still numerous issues to delve into, like minimum amount of cell content, variant classification, and clinical reporting of incidental or novel, and potential actionable, mutations (11, 12). Although the use and standardization of targeted NGS is still in early development, a large ongoing study for differentiating malignant from benign thyroid nodules from fine-needle aspirates, based on NGS analysis of mutations and gene fusions associated with most thyroid cancers, have shown promising results and may prevent surgeries (13).

In broad, efforts regarding somatic mutations and potential targeted therapy have naturally focused on patients with advanced cancers, where standard therapy was no longer effective (14). Yet, many targetable mutations are already present at the time of primary diagnosis and may potentially guide the clinical course on a personalized level, especially when larger studies with clinical follow-up emerge. Noteworthily, a recent translational study correlated the somatic signature of 39 FTC to both diagnosis and prognosis and found that mutation burden was associated to a worse prognosis, independent of histopathological classification (15). The study did not show a clear correlation between somatic events and the three morphology-based FTC subtypes: minimally invasive, encapsulated angioinvasive, and widely invasive.

Here, two independent amplicon-based NGS-panels were applied to identify and validate the somatic mutation profile in a sample-set of follicular cell-derived thyroid neoplasia. All mutations were classified according to recent guidelines (16). Hence, the somatic mutation fingerprint of the FTCs-spectrum, from FA to the anaplastic thyroid cancer, were assigned to the morphology and IHC-based, “gold-standard” diagnostic classes to explore the correlation and assess if these heterogenous FTCs could, to some extent, be classified according to molecular markers and support a more quantitative and objective approach.

## Materials and Methods

### Samples

Archived formalin fixed paraffin embedded (FFPE) tissue samples (*n* = 101) were collected for downstream NGS analysis originating from ATC (*n* = 7), PDC (*n* = 7), wiFTC (*n* = 11), miFTC (*n* = 19), PTC (*n* = 24), and FA (*n* = 33). The follicular diagnostic groups did not include oncocytic and clear cell type, and as for the papillary diagnostic group, tall cell and follicular variant subtypes were excluded. Prior current molecular analysis, FFPE samples were kept at room temperature at the Department of Pathology, Copenhagen University Hospital. Samples were anonymized prior to DNA extraction, according to national legislation and institutional requirements, thus waiving the requirement for written informed consent. The study is a technical and quality assessment of high-throughput sequencing platforms for somatic variant identification as a part of the project (no. H-4-2014-039) approved by the ethics committee of the Capital Region of Denmark and Data Protection Agency (no. HEH-2014-016, I-Suite no: 02657).

### Illumina Workflow

DNA extraction was performed from punched samples from FFPE tissue blocks, circumventing stromal and necrotic tissue, using the Maxwell 16 FFPE Tissue LEV DNA Purification Kit, performed according to the manufacturer's instructions. We used 250 ng of each DNA sample with the TruSeq Amplicon Cancer Panel (Illumina, San Diego, California, USA) according to the manufacturer's instructions. The TruSeq Amplicon Cancer Panel comprises 48 genes; *ABL1, AKT1, ALK, APC, ATM, BRAF, CDH1, CDKN2A, CSF1R, CTNNB1, EGFR, ERBB2, ERBB4, FBXW7, FGFR1, FGFR2, FGFR3, FLT3, GNA11, GNAQ, GNAS, HNF1A, HRAS, IDH1, JAK2, JAK3, KDR, KIT, KRAS, MET, MLH1, MPL, NOTCH1, NPM1, NRAS, PDGFRA, PIK3CA, PTEN, PTPN11, RB1, RET, SMAD4, SMARCB1, SMO, SRC, STK11, TP53*, and *VHL*. After PCR cleanup, library quality was assessed on a 2,100 Bioanalyzer (Agilent Technologies, Santa Clara, USA). Instead of bead normalization, the libraries were quantified using the Qubit dsDNA BR assay (Life Technologies, Grand Island, New York, USA) and diluted to 4 nM with 10 mM Tris-HCl pH 8.5 supplemented with 0.1% Tween 20. Libraries were prepared for sequencing according to the standard normalization method described in the “MiSeq System Denature and Dilute Libraries Guide.” An addition of 10 μl 200 mM Tris-HCl pH 7 was made after denaturation. Paired-end sequencing was performed using MiSeq Reagent Kit v2, 500 cycles. Data were aligned and analyzed using CLC Genomic Workbench, and variants were called at a minimum frequency of 10%. Common variants were excluded using the software Ingenuity Variant Analysis (IVA; http://ingenuity.com). Subsequently, all variants were manually assessed to ensure no false positive mutations in the final curated list. The sequencing data is deposited in EMBL European Variation Archive database (Accession Number: PRJEB36753).

### Qiagen Workflow-Validation

Depending on the size of the embedded tissue, DNA was extracted from two to five 10 μm thick sections of each FFPE sample using the GeneRead DNA FFPE Kit (Qiagen). Proteinase K digestion was performed using twice the recommended volume of water, buffer FTB and enzyme, and the incubation at 56°C was carried out overnight. The bind, wash and elute steps were performed on a QIAcube robotic workstation (Qiagen). The extracted DNA was quantified using a QIAxpert UV spectrophotometer (Qiagen). The Actionable Insights Tumor Panel and GeneRead DNAseq Panel PCR Kit V2 comprising *KRAS, NRAS, KIT, BRAF, PDGFRA, ALK, EGFR, ERBB2, PIK3CA, ERBB3, ESR1*, and *RAF1* were used for target enrichment. Following the manufacturer's instructions, a total of 40 ng per DNA sample were divided into four multiplex PCR pools. After PCR cleanup, quality assessment was made on a 2,100 Bioanalyzer and quantified on the QIAxpert. Library construction using 30 ng of target enriched DNA and the GeneRead DNA Library Q Kit, was performed on the QIAcube. Final library quality was assessed on the Bioanalyzer and quantified using the QIAxpert. The GeneRead Clonal Amp Q kit was used to prepare sequencing templates by clonal amplification according to the manufacturer's instructions. After library normalization and pooling, a final pool concentration of 2 pg/μl was used for the emulsion making. Emulsion making, breaking and pooling, as well as bead enrichment, were performed on a GeneRead QIAcube (Qiagen). Determination of bead concentration was made using OD measurement on the QIAxpert as described in the protocol. The GeneRead Sequencing Q Kit was used for preparing and running the loaded flow cell on the GeneReader. Data were aligned and analyzed using the build in analyzing tool based on Qiagen biomedical Genomics workbench, variants were called down to a frequency of 10%. To have comparable datasets the variant files (VCF) were exported and used for analysis in Ingenuity. In Ingenuity the variants were filtered so the final list of variants only included not common variants.

### Variant Classification

The possible diagnostic and therapeutic relevance of the individually curated variants were further assessed according to recent international guidelines for somatic variant classification (11). According to the guidelines the variants are assigned into four tiers (I-IV) based on the level of clinical significance in cancer diagnosis, prognosis, and/or therapeutic relevance. Tier I: variants with strong clinical significance; II: variants with potential clinical significance; III: variants with unknown clinical significance; IV: benign or likely benign variants.

### Clustering Analysis

Clustering analysis was performed in R version 3.4.1, using the default parameter of the “heatmap 3” R-package. The clustering was performed on the number of mutations per gene per sample, indicated by the color intensity in **Figure 3**. The samples were clustered based on dissimilarities of the mutational profile defined as number of mutations per gene. Furthermore, the genes were clustered according to number of and co-occurrence of mutations in the different samples. The samples were labeled according to the diagnostic groups (ATC, PDC, wiFTC, miFTC, PTC, and FA) in the heatmap. Subsequent to the clustering of the samples, they were assigned to mutational signatures (A through D) based on similarity to other samples with regards to the mutational load and which genes were mutated.

## Results

**Figure 1:**
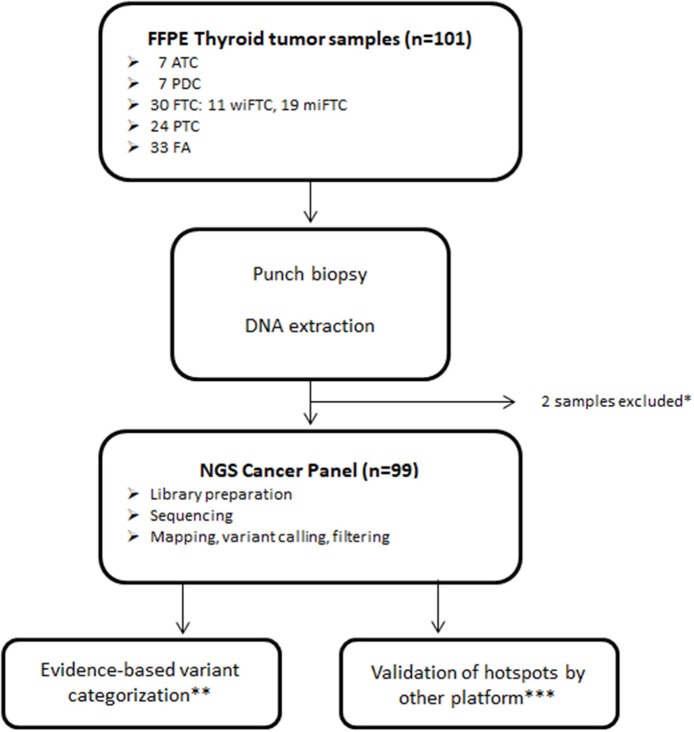
Sample and sequencing pipeline resulting in 99 samples for downstream analysis. *Samples excluded due to insufficient DNA yield; **Classified according to Tier categorization recommended by Li et al. (11); ***Validation of selected mutations was performed using the GeneRead Clonal Amp Q kit on the Genereader platform.

**Figure 2:**
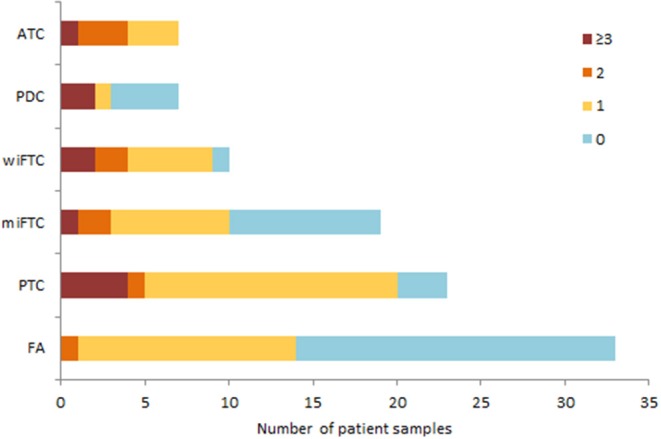
The bar plot illustrates mutation burden per sample according to diagnostic group. Mutation burden is defined as the sum of mutations, regardless of gene, and categorized as 0 (blue); 1 (yellow); 2 (orange) or ≥3 (red) mutations in total.

Archived FFPE samples from 101 patients diagnosed with follicular cell-derived neoplasia were included and analyzed according to the pipeline depicted in Figure 1. DNA extracted from punch biopsies from the tumor resulted in sufficient DNA-amount and -quality for downstream NGS-analysis of 99 samples (ATC = 7; PDC = 7; wiFTC = 10; miFTC = 19; PTC = 23; FA = 33). All samples were sequenced with mean coverage of 7199x [4025-14989x]. Mutations were identified in the 48 hotspot genes in 63 of the sequenced samples and none in the remaining 36 (further detailed in Supplementary Table 1). Two samples were excluded prior to DNA-library preparation, as 99.98% passed the DNA quality criterion. The distribution of mutations in the specific diagnostic groups resulted in a higher mutation frequency among the most dedifferentiated groups (ATC, PDC, wiFTC) than in the groups with a more differentiated cell profile. These results are illustrated as number of mutations per sample in Figure 2. Notably, 42% (14/33) of the benign samples (FA) harbored a mutation in one of the oncogenes. In the miFTC group 47% (9/19) of the samples did not have any mutations in the analyzed oncogenes. In addition, more than half (57%) of the PDC (4/7) samples did not harbor a mutation, although this group is highly malignant.

**Figure 3:**
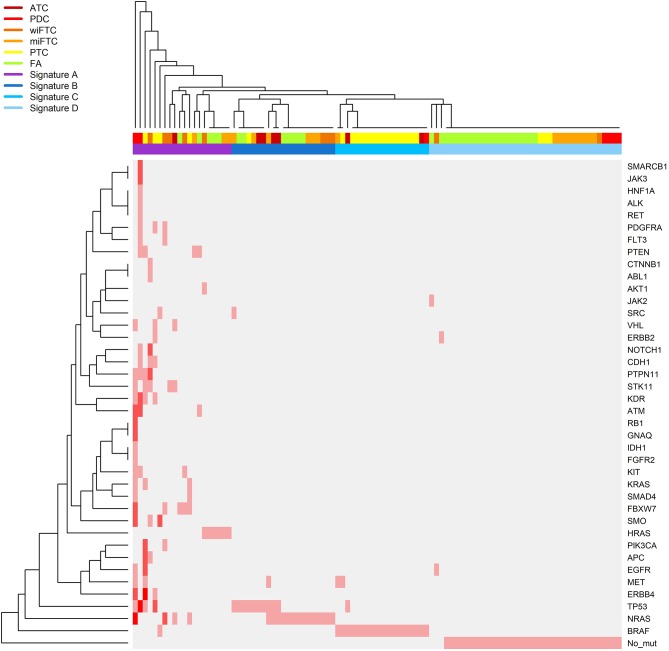
Heatmap illustrating a hierarchical clustering of samples according to the number of mutations identified in the gene panel. The samples follow the X-axis and are clustered according to their mutational pattern. Genes in which mutations were identified are listed on the Y-axis (“No_mut” corresponds to no identified mutations in any of the sequenced genes), the genes are clustered according to the number of and co-occurrence of mutations in the samples. The relative color scheme in the plot indicates the number of mutations in sample and gene: pale gray indicates no mutations, and red corresponds to the maximum number of identified mutations. The top color bar on the X-axis specifies the IHC class: ATC, dark red; PDC, red; wiFTC, dark orange; miFTC, orange; PTC, yellow; FA, green; and the mutational signatures; Signature A, highly mutated samples (purple); Signature B, mainly *NRAS* and *TP53* mutations (dark blue); Signature C, *BRAF* (blue); Signature D, none or single sporadic mutations (light blue).

Next, we sought to determine which of the oncogenes were the most frequently mutated across the different diagnostic groups (complete list of mutations in Supplementary Table 1). Figure 3 shows a heatmap of hierarchical clustering of genes according to number of mutations per sample and annotated to diagnostic groups. Furthermore, the samples are clustered according to the pattern of mutated genes. Close to 70% (16 of the 23 samples) of the PTC samples harbored a *BRAF*-mutation (V600E), followed by *TP53* (T230P, L257P, R282L, E286D) and *ERBB4* (C258R, C234Y, C308Y, I929T) mutated in 17% (four samples), respectively. The most frequently mutated genes in FA, miFTC, wiFTC, and ATC samples were *NRAS* (G13R, D38N, T58R, Q61K, Q61R, E62K), *HRAS* (Q61K, Q61R), and *TP53* (S127F, N131del, P152S, A161T, Y163C, Y163N, G244V, M237I, R209Kfs^*^6, c.673-2A>T). In the PDC samples the most frequently mutated genes were: *TP53* (57%, 4 samples; Q5R, Q167L, V172F, E286K), *ATM* (57%, 4 samples; K1323I, T2666I, S3027I, P3050L)*, KDR* (43%, 3 samples; V226M, E987G, V1318L), and *NRAS* (43%, 3 samples; S17G, A59S, A66P). In the wiFTC samples the most frequently mutated gene was *NRAS* (50%, 5 samples; T58R, Q61K, Q61R). A visual interpretation of the heatmap resulted in four mutational signatures (Signature A through D) according to the sample clustering; Signature A: highly mutated samples, Signature B: mainly *NRAS* and *TP53* mutations, Signature C: *BRAF*, Signature D: none or single sporadic mutations. The sample clustering based on the somatic mutations found in the samples, and the subsequent assignment of mutational signatures highlights the heterogeneity within and among the diagnostic IHC classification.

**Figure 4:**
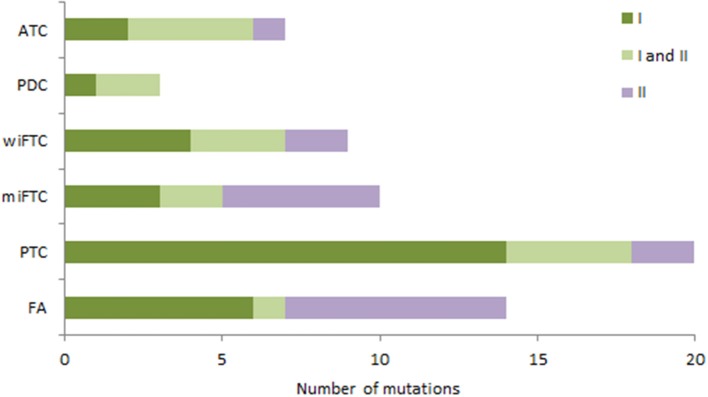
The bar plot illustrates the distribution of tier classified mutations in the diagnostic groups (samples without mutations are not included). Mutations are assigned according to Tier classification scheme; samples with only Tier I mutations (dark green); samples with both Tier I and II mutations (light green); samples with only Tier II mutations (purple).

To assess the clinical relevance of the identified mutations across the different diagnostic groups we qualified each mutation's therapeutic, prognostic and diagnostic evidence level according to the recent guidelines for somatic mutations (11). The classification of variants resulted in only Tier I and II since the sequenced Genes originates from pre-defined cancer gene panels, which is why the criteria for Tier III and IV were not applicable. Figure 4 depicts the distribution of Tier I and/or II among the mutation-positive samples in the diagnostic groups. Most of the samples had one or more Tier I mutations, reflecting a strong clinical significance with either an FDA-approved targeted therapy available or consensus of a clinical trial enrollment based on molecular targets. As illustrated in Figure 4, the malignant subgroups had >78% mutations with a strong clinical significance (Tier I) apart from the miFTC where only 50% harbored Tier I mutations, resembling the distribution in the benign group (FA).

## Discussion

Although progress has been made toward characterizing the molecular portrait of follicular cell derived carcinomas, there is little advancement in the association of the mutational signature to the WHO diagnostic classes. Apart from the majority of *BRAF*-positive PTCs, the frequency of potentially targetable mutations in primary thyroid cancers are relative unexploited. We set out to deep sequence a large set of primary follicular cell derived carcinomas to identify and classify somatic mutations in a preselected set of pan cancer genes.

Overall, sequencing of hot spot genes in archived FFPE blocks from follicular cell derived neoplasia showed that the number of mutations followed the grade of malignancy across the different diagnostic groups. However, in more than half of the PDCs, which is assigned as a highly malignant tumor type, we did not identify any hot-spot gene mutations. This could be due to mutations in genes not included in the hot-spot panel, or the malignant behavior of PDC could be caused by larger chromosomal aberrations not detectable with the analytic technique used in the present study. In a recent study where 23 PDCs were screened for somatic mutations using a similar approach to ours, 80% harbored potentially pathogenic mutations (17), thus a portion of PDCs either do not harbor somatic point mutations or variants are not covered with a pre-defined cancer panel. Noteworthy, in several of the FAs (42%), we did identify mutations, where a few, in fact, are known hot-spot mutations e.g., *NRAS* Q61R, activating the Mapk signaling pathway (18). This result strongly supports the findings by Borowczyk et al. (19) who recently identified potentially pathogenic mutations in 40% of FAs using a similar sequencing approach. Also, among the FTCs there is a striking agreement as Borowczyk et al. (19) found 69% harboring potentially pathogenic mutations as we found 67% when merging miFTC and wiFTC into one subtype. The similarity of the somatic mutation profiles among FAs and FTCs supports the point raised regarding FTCs more likely being a continuum of FAs rather than a distinct diagnostic entity and will probably add to the ongoing scientific discussion (20, 21). Recent studies have explored the possible correlation of the somatic mutation signature and burden to histopathological diagnostic classes and found no substantial correlation (2, 15, 22, 23). However, two of the studies succeeded in showing a significant correlation between patient prognosis and mutational signature and/or overall mutation load (15, 23). In brief, the study by Nicolson et al. (15) correlated the somatic signature of 39 FTC to both diagnosis and prognosis and found that mutation burden was associated to a worse prognosis, independent of histopathological classification. The study did not show a clear correlation between somatic events and the three morphology-based classes; miFTC, wiFTC, and encapsulated angioinvasive FTC subtypes, however this could be due to a limited sample number or that the morphology-based diagnosis does not entirely reflect the mutational signature. The sequencing method used by Nicolson et al. (15) was whole exome sequencing (WES) which allow for identification of mutations in all coding regions. Nonetheless, WES is not suitable for capturing low frequent mutations, e.g., subclonal entities, due to limited read depths. Moreover, WES does not encompass calling of other cancer related molecular mechanisms, like copy number alterations and epigenetic changes.

In the present study, we used a pre-defined set of genes, leaving a potential risk of overlooking driver mutations in other genes. Hence, some of our mutation negative samples may plausibly be false-negative, which may explain the lack of identified mutations in some of the samples within the dedifferentiated diagnostic groups. However, the advantage of our pre-defined pan cancer panel is high coverage enabling identification of low frequent driver mutations. We did indeed take advantage of the high coverage of more than 4000x and identified several mutations in e.g., *NRAS, TP53, BRAF*, and *ALK* with an allele frequency just above the 10% cut-off. Moreover, the pre-defined gene panel enabled us to perform manual curation of all called mutations and assess their clinical significance according to international variant classification guidelines for somatic events. The mutations were further verified using an independent sequencing platform to ensure true positive results.

The clinical relevance of the most frequently mutated genes in follicular cell derived carcinomas, e.g., *BRAF, TP53*, and *NRAS* is still not uniform; BRAFV600E occurs in about 29–83% of cases and is the most common molecular aberration in thyroid cancer (24) and is frequently associated with tumor aggressiveness and poor prognosis due to a constitutive activation of the mitogen-activated protein kinase 1 (MAPK) signaling pathway. Agents targeting thyroid cancers harboring the BRAFV600F mutation has been approved for metastatic thyroid cancer. However, early trials have shown a significant effect on progression-free-survival, a positive benefit on overall survival is still not evident (25).

Overall, there is a lack of standardization regarding somatic variant classification among clinical pathologists, oncologists, molecular biologists and in the scientific literature in general. Therefore, we assigned all the variants with a class based on the recent Tier classification model (11). However, since we used a pre-defined cancer gene panel, only Tier I and II were applicable. The difference between Tier I and II is highly relevant, since only Tier I variants (variants with strong clinical significance) are known pathogenic somatic mutations, whereas Tier II (variants with potential clinical significance) are merely variants of unknown significance although they are present in a cancer-related gene. Among the FAs we found 40% mutation positive (Tier I and II), yet only 18% (6/33) were, in fact, known hot-spot mutations of strong clinical relevance (Tier I), see Figure 4. Among the wiFTCs close to all the nine samples that were mutation positive harbored at least one Tier I variant (7/9, see Supplementary Table 1). Thus, the Tier-scheme may add a more qualified assessment of the variant interpretation, but indeed a more comprehensive validation of the recent Tier classification scheme is necessary.

We assessed the mutational pattern over the entire sample set and carefully suggested four mutational signatures from A to D where A is characterized by a high frequency of mutations per samples, B mainly by *NRAS* and *TP53* mutations, C by *BRAF* positive samples and D by none or only single sporadic mutations. These signatures can certainly not stand alone and are limited by the lack of clinical follow-up data, not applicable for the present study. However, since the amplicon based NGS panel is almost a 100% compatible (99.98% passed the DNA quality criterion prior to DNA-library preparation), this urges for a prospective study with long-term follow-up of the clinical course. This could result in distinct mutational signatures to support and expand the morphological based classification scheme and ultimately identify patients for different therapeutic groups consisting of targeted therapy.

## Data Availability Statement

The sequencing data is deposited in EMBL European Variation Archive database (Accession Number: PRJEB36753). The raw data supporting the conclusions of this article will be made available by the authors, without undue reservation, to any qualified researcher.

## Ethics Statement

Samples were anonymized prior to DNA extraction, according to national legislation and institutional requirements, thus The Ethics committee of the Capital Region of Denmark waived the requirement for written informed consent. The study is a technical and quality assessment of high-throughput sequencing platforms for somatic variant identification as a part of the project (No. H-4-2014-039) approved by the ethics committee of the Capital Region of Denmark and Data Protection Agency (No. HEH-2014-016, I-Suite No: 02657).

## Author Contributions

All authors listed have made a substantial, direct and intellectual contribution to the work, and approved it for publication.

### Conflict of Interest

The authors declare that the research was conducted in the absence of any commercial or financial relationships that could be construed as a potential conflict of interest.
