# Supplementary material for: Amplicon-Based NGS Panels for Actionable Cancer Target Identification in Follicular Cell-Derived Thyroid Neoplasia
Source: Front Endocrinol (Lausanne). 2020 Mar 24;11:146. doi: 10.3389/fendo.2020.00146 (PMC7105679; doi:10.3389/fendo.2020.00146)
Supplement: Supplementary file 1 [file Table_1.pdf]

Madsen MB, Kiss K, Cilius Nielsen F, Bennedbæk FN and Rossing M (2020). Amplicon-Based NGS Panels for Actionable Cancer Target Identification in Follicular Cell-Derived Thyroid Neoplasia. Front. Endocrinol. 11:146. doi: 10.3389/fendo.2020.00146

Supplementary Table 1.

Identified mutations in the 99 studied samples listed below, along with call frequency, tier classification and mutational signature (signature A: highly mutated samples, signature B: mainly *NRAS* and *TP53* mutations, signature C: *BRAF*, signature D: none or single sporadic mutations).

Sample number	Diagnostic group	Mutational signature	Gene	Nucleotide change	Amino acid change	Call freqency in percent	Tier classification
**1**	ATC	Signature B	*TP53*	c.626_627delGA	R209Kfs*6	29	I
**2**	ATC	Signature B	*NRAS*	c.37G>C	G13R	31	I
**2**	ATC	Signature B	*TP53*	c.487T>A	Y163N	27	II
**3**	ATC	Signature C	*BRAF*	c.1799T>A	V600E	28	I
**4**	ATC	Signature A	*NRAS*	c.112G>A	D38N	10	II
**4**	ATC	Signature A	*STK11*	c.248delA	K83Rfs*13	20	I
**4**	ATC	Signature A	*VHL*	c.308C>T	P103L	18	II
**5**	ATC	Signature C	*BRAF*	c.1799T>A	V600E	36	I
**5**	ATC	Signature C	*TP53*	c.454C>T	P152S	34	II
**6**	ATC	Signature B	*TP53*	c.731G>T	G244V	79	II
**7**	ATC	Signature B	*NRAS*	c.182A>G	Q61R	36	I
**7**	ATC	Signature B	*TP53*	c.380C>T	S127F	42	II
**8**	PDC	Signature A	*ALK*	c.3824G>A	R1275Q	10	I
**8**	PDC	Signature A	*ATM*	c.9080G>T	S3027I	12	II
**8**	PDC	Signature A	*ATM*	c.7997C>T	T2666I	11	II
**8**	PDC	Signature A	*CDH1*	c.1195A>G	T399A	18	II
**8**	PDC	Signature A	*FLT3*	c.1829dupT	L610Ffs*24	19	II
**8**	PDC	Signature A	*HNF1A*	c.622C>T	P208S	12	II
**8**	PDC	Signature A	*JAK3*	c.1730dupG	S577Rfs*85	11	II
**8**	PDC	Signature A	*JAK3*	c.2143G>A	V715I	20	II
**8**	PDC	Signature A	*KDR*	c.2960A>G	E987G	23	II
**8**	PDC	Signature A	*KDR*	c.3952G>C	V1318L	11	II
**8**	PDC	Signature A	*KIT*	c.1957A>T	I653F	19	II
**8**	PDC	Signature A	*NOTCH1*	c.4678delG	D1560Tfs*20	39	II
**8**	PDC	Signature A	*PDGFRA*	c.1709A>G	H570R	19	II
**8**	PDC	Signature A	*PTEN*	c.524T>C	V175A	26	I
**8**	PDC	Signature A	*PTPN11*	c.239A>G	Y80C	23	I
**8**	PDC	Signature A	*RET*	c.1975delT	C659Afs*16	40	II
**8**	PDC	Signature A	*SMARCB1*	c.200C>T	S67L	11	II
**8**	PDC	Signature A	*SMARCB1*	c.130T>C	Y44H	12	II
**8**	PDC	Signature A	*TP53*	c.500A>T	Q167L	15	II
**8**	PDC	Signature A	*TP53*	c.514G>T	V172F	14	I
**8**	PDC	Signature A	*TP53*	c.856G>A	E286K	23	II
**9**	PDC	Signature D	No mutation	NA	NA	NA	NA
**10**	PDC	Signature D	No mutation	NA	NA	NA	NA
**11**	PDC	Signature C	*BRAF*	c.1799T>A	V600E	35	I

**12**	PDC	Signature A	*ATM*	c.9149C>T	P3050L	51	II
**12**	PDC	Signature A	*ATM*	c.3968A>T	K1323I	24	II
**12**	PDC	Signature A	*EGFR*	c.2569G>T	G857W	38	I
**12**	PDC	Signature A	*ERBB4*	c.662G>A	C221Y	42	II
**12**	PDC	Signature A	*ERBB4*	c.1718dupG	P574Sfs*2	32	II
**12**	PDC	Signature A	*FBXW7*	c.794T>A	V265E	11	II
**12**	PDC	Signature A	*FBXW7*	c.1183G>T	V395F	39	II
**12**	PDC	Signature A	*FGFR2*	c.1133delT	S372Pfs*7	25	II
**12**	PDC	Signature A	*GNAQ*	c.748G>C	E250Q	23	II
**12**	PDC	Signature A	*GNAQ*	c.913G>A	A305T	21	II
**12**	PDC	Signature A	*IDH1*	c.328G>A	E110K	17	I
**12**	PDC	Signature A	*KDR*	c.676G>A	V226M	23	II
**12**	PDC	Signature A	*KIT*	c.2162A>G	Y721C	12	II
**12**	PDC	Signature A	*KRAS*	c.366delT	R123Efs*3	18	II
**12**	PDC	Signature A	*MET*	c.3820C>T	Q1274*	21	II
**12**	PDC	Signature A	*NRAS*	c.175G>T	A59S	12	II
**12**	PDC	Signature A	*NRAS*	c.49A>G	S17G	43	II
**12**	PDC	Signature A	*NRAS*	c.196G>C	A66P	15	II
**12**	PDC	Signature A	*PTPN11*	c.1576C>T	Q526*	20	I
**12**	PDC	Signature A	*RB1*	c.1123G>T	V375F	14	II
**12**	PDC	Signature A	*RB1*	c.388A>T	K130*	16	II
**12**	PDC	Signature A	*SMAD4*	c.1094G>A	G365D	15	II
**12**	PDC	Signature A	*SMO*	c.1086delG	L363Sfs*24	40	I
**12**	PDC	Signature A	*SMO*	c.965A>C	T322S	14	I
**12**	PDC	Signature A	*STK11*	c.631C>T	R211W	21	I
**12**	PDC	Signature A	*TP53*	c.14A>G	Q5R	20	II
**12**	PDC	Signature A	*VHL*	c.416C>T	S139F	11	II
**13**	PDC	Signature D	No mutation	NA	NA	NA	NA
**14**	PDC	Signature D	No mutation	NA	NA	NA	NA
**15**	wiFTC	Signature A	*ABL1*	c.1082A>G	H361R	13	II
**15**	wiFTC	Signature A	*APC*	c.4241delT	V1414fs	14	I
**15**	wiFTC	Signature A	*CDH1*	c.360delG	H121Tfs*94	31	II
**15**	wiFTC	Signature A	*CTNNB1*	c.14-2A>G		34	II
**15**	wiFTC	Signature A	*NOTCH1*	c.4696C>G	P1566A	17	II
**15**	wiFTC	Signature A	*NOTCH1*	c.4778T>A	L1593Q	15	II
**15**	wiFTC	Signature A	*PTPN11*	c.232delG	V78Sfs*11	20	I
**15**	wiFTC	Signature A	*PTPN11*	c.212T>C	F71S	20	I
**15**	wiFTC	Signature A	*SMO*	c.1092delC	W365Gfs*22	18	I
**15**	wiFTC	Signature A	*STK11*	c.842delC	P281Rfs*6	11	I
**16**	wiFTC	Signature B	*NRAS*	c.182A>G	Q61R	47	I
**17**	wiFTC	Signature A	*STK11*	c.157_158insT	D53Vfs*110	42	I
**18**	wiFTC	Signature D	*EGFR*	c.1852C>T	H618Y	11	II
**19**	wiFTC	Signature A	*FBXW7*	c.1717T>A	C573S	11	II
**19**	wiFTC	Signature A	*FLT3*	c.1717G>A	E573K	13	II
**19**	wiFTC	Signature A	*NRAS*	c.173C>G	T58R	34	II
**19**	wiFTC	Signature A	*NRAS*	c.182A>G	Q61R	34	I
**19**	wiFTC	Signature A	*PDGFRA*	c.1988C>T	A663V	14	II
**19**	wiFTC	Signature A	*PIK3CA*	c.1045C>T	R349*	10	II

**20**	wiFTC	Signature A	*FBXW7*	c.779G>A	C260Y	11	II
**20**	wiFTC	Signature A	*KIT*	c.2141+2T>A		10	II
**21**	wiFTC	Signature B	*NRAS*	c.182A>G	Q61R	31	I
**22**	wiFTC	Signature A	*AKT1*	c.49G>A	E17K	16	I
**22**	wiFTC	Signature A	*HRAS*	c.182A>G	Q61R	44	II
**23**	wiFTC	Signature D	No mutation	NA	NA	NA	NA
**24**	wiFTC	Signature B	*NRAS*	c.181C>A	Q61K	44	I
**25**	miFTC	Signature D	No mutation	NA	NA	NA	NA
**26**	miFTC	Signature D	No mutation	NA	NA	NA	NA
**27**	miFTC	Signature D	No mutation	NA	NA	NA	NA
**28**	miFTC	Signature B	*NRAS*	c.182A>G	Q61R	39	I
**29**	miFTC	Signature B	*SRC*	c.1598G>A	G533E	10	II
**29**	miFTC	Signature B	*TP53*	c.673-2A>T		21	II
**30**	miFTC	Signature D	No mutation	NA	NA	NA	NA
**31**	miFTC	Signature B	*NRAS*	c.182A>G	Q61R	44	I
**32**	miFTC	Signature B	*MET*	c.2975C>T	T992I	16	II
**32**	miFTC	Signature B	*NRAS*	c.182A>G	Q61R	10	I
**32**	miFTC	Signature B	*TP53*	c.711G>A	M237I	27	I
**33**	miFTC	Signature B	*TP53*	c.481G>A	A161T	45	II
**34**	miFTC	Signature D	No mutation	NA	NA	NA	NA
**35**	miFTC	Signature A	*HRAS*	c.182A>G	Q61R	42	II
**36**	miFTC	Signature A	*PTEN*	c.611C>T	P204L	12	II
**37**	miFTC	Signature D	No mutation	NA	NA	NA	NA
**38**	miFTC	Signature D	No mutation	NA	NA	NA	NA
**39**	miFTC	Signature C	*BRAF*	c.1801A>G	K601E	41	I
**39**	miFTC	Signature C	*MET*	c.3029C>T	T1010I	50	II
**40**	miFTC	Signature D	No mutation	NA	NA	NA	NA
**41**	miFTC	Signature B	*NRAS*	c.182A>G	Q61R	51	I
**42**	miFTC	Signature D	No mutation	NA	NA	NA	NA
**43**	miFTC	Signature A	*HRAS*	c.182A>G	Q61R	37	II
**44**	PTC	Signature C	*BRAF*	c.1799T>A	V600E	25	I
**45**	PTC	Signature C	*BRAF*	c.1799T>A	V600E	40	I
**45**	PTC	Signature C	*MET*	c.2975C>T	T992I	49	II
**46**	PTC	Signature A	*FBXW7*	c.1397G>A	C466Y	15	II
**46**	PTC	Signature A	*KRAS*	c.158T>A	L53*	25	II
**46**	PTC	Signature A	*NRAS*	c.181C>A	Q61K	23	I
**46**	PTC	Signature A	*SMAD4*	c.1543A>T	R515*	13	II
**47**	PTC	Signature C	*BRAF*	c.1799T>A	V600E	30	I
**48**	PTC	Signature C	*BRAF*	c.1799T>A	V600E	43	I
**49**	PTC	Signature C	*BRAF*	c.1799T>A	V600E	40	I
**50**	PTC	Signature C	*BRAF*	c.1799T>A	V600E	41	I
**51**	PTC	Signature C	*BRAF*	c.1799T>A	V600E	40	I
**52**	PTC	Signature C	*BRAF*	c.1799T>A	V600E	23	I
**53**	PTC	Signature C	*BRAF*	c.1799T>A	V600E	39	I

**54**	PTC	Signature C	*BRAF*	c.1799T>A	V600E	40	I
**55**	PTC	Signature C	*BRAF*	c.1799T>A	V600E	39	I
**56**	PTC	Signature A	*BRAF*	c.1799T>A	V600E	12	I
**56**	PTC	Signature A	*SMO*	c.1903delC	Q635Rfs*141	12	I
**56**	PTC	Signature A	*SMO*	c.1216G>C	A406P	26	I
**56**	PTC	Signature A	*SRC*	c.1610A>C	*537Sext*60	15	II
**57**	PTC	Signature D	No mutation	NA	NA	NA	NA
**58**	PTC	Signature C	*BRAF*	c.1799T>A	V600E	30	I
**59**	PTC	Signature D	No mutation	NA	NA	NA	NA
**60**	PTC	Signature C	*BRAF*	c.1799T>A	V600E	19	I
**61**	PTC	Signature A	*CDH1*	c.1256A>T	D419V	15	II
**61**	PTC	Signature A	*ERBB2*	c.2645G>A	G882D	31	II
**61**	PTC	Signature A	*ERBB4*	c.772T>C	C258R	17	II
**61**	PTC	Signature A	*KDR*	c.2971+1G>A		27	II
**61**	PTC	Signature A	*PDGFRA*	c.1661G>T	R554M	34	II
**61**	PTC	Signature A	*TP53*	c.688A>G	T230P	33	II
**61**	PTC	Signature A	*TP53*	c.770T>C	L257P	34	II
**61**	PTC	Signature A	*VHL*	c.408T>A	F136L	10	II
**62**	PTC	Signature D	No mutation	NA	NA	NA	NA
**63**	PTC	Signature C	*BRAF*	c.1799T>A	V600E	15	I
**64**	PTC	Signature A	*APC*	c.4666dupA	T1556fs	13	I
**64**	PTC	Signature A	*APC*	c.3416delA	L1139fs	12	I
**64**	PTC	Signature A	*EGFR*	c.2176G>A	V726M	17	II
**64**	PTC	Signature A	*EGFR*	c.361G>T	E1206*	12	II
**64**	PTC	Signature A	*ERBB4*	c.701G>A	C234Y	12	I
**64**	PTC	Signature A	*ERBB4*	c.923G>A	C308Y	16	II
**64**	PTC	Signature A	*ERBB4*	c.2786T>C	I929T	19	II
**64**	PTC	Signature A	*KDR*	c.3641delA	Y1214Lfs*21	17	II
**64**	PTC	Signature A	*KRAS*	c.179G>A	G60D	13	II
**64**	PTC	Signature A	*MET*	c.481A>G	I161V	11	II
**64**	PTC	Signature A	*PIK3CA*	c.3040C>T	Q1014*	12	II
**64**	PTC	Signature A	*PIK3CA*	c.1034A>G	N345S	14	II
**64**	PTC	Signature A	*PTEN*	c.562T>A	Y188N	15	II
**64**	PTC	Signature A	*PTPN11*	c.1538T>A	F513Y	12	I
**64**	PTC	Signature A	*STK11*	c.246G>T	K82N	20	I
**64**	PTC	Signature A	*TP53*	c.858A>T	E286D	11	II
**65**	PTC	Signature C	*BRAF*	c.1799T>A	V600E	45	I
**66**	PTC	Signature B	*TP53*	c.845G>T	R282L	50	II
**67**	FA	Signature D	No mutation	NA	NA	NA	NA
**68**	FA	Signature D	*ERBB2*	c.2417T>A	L806H	23	II
**69**	FA	Signature B	*NRAS*	c.182A>G	Q61R	48	I
**70**	FA	Signature B	*TP53*	c.488A>G	Y163C	32	I
**71**	FA	Signature A	*ATM*	c.9103C>T	L3035F	79	II
**71**	FA	Signature A	*PTEN*	c.655C>T	Q219*	54	I
**72**	FA	Signature D	No mutation	NA	NA	NA	NA
**73**	FA	Signature A	*HRAS*	c.182A>G	Q61R	40	II

**74**	FA	Signature D	No mutation	NA	NA	NA	NA
**75**	FA	Signature D	No mutation	NA	NA	NA	NA
**76**	FA	Signature B	*NRAS*	c.182A>G	Q61R	41	I
**77**	FA	Signature A	*HRAS*	c.181C>A	Q61K	44	II
**78**	FA	Signature B	*NRAS*	c.182A>G	Q61R	44	I
**79**	FA	Signature A	*FBXW7*	c.1318G>A	D440N	19	II
**80**	FA	Signature B	*TP53*	c.393_395delCAA	N131del	85	II
**81**	FA	Signature D	No mutation	NA	NA	NA	NA
**82**	FA	Signature D	No mutation	NA	NA	NA	NA
**83**	FA	Signature D	No mutation	NA	NA	NA	NA
**84**	FA	Signature D	No mutation	NA	NA	NA	NA
**85**	FA	Signature D	No mutation	NA	NA	NA	NA
**86**	FA	Signature D	No mutation	NA	NA	NA	NA
**87**	FA	Signature D	No mutation	NA	NA	NA	NA
**88**	FA	Signature D	No mutation	NA	NA	NA	NA
**89**	FA	Signature D	No mutation	NA	NA	NA	NA
**90**	FA	Signature B	*NRAS*	c.182A>G	Q61R	39	I
**91**	FA	Signature D	*JAK2*	c.2164G>A	E722K	54	II
**92**	FA	Signature D	No mutation	NA	NA	NA	NA
**93**	FA	Signature B	*NRAS*	c.184G>A	E62K	12	II
**94**	FA	Signature D	No mutation	NA	NA	NA	NA
**95**	FA	Signature D	No mutation	NA	NA	NA	NA
**96**	FA	Signature D	No mutation	NA	NA	NA	NA
**97**	FA	Signature D	No mutation	NA	NA	NA	NA
**98**	FA	Signature A	*HRAS*	c.182A>G	Q61R	32	II
**99**	FA	Signature D	No mutation	NA	NA	NA	NA
